# Circular RNAs as Trojan horses of leukaemia: A promising path to cancer precision medicine

**DOI:** 10.1002/ctm2.1469

**Published:** 2023-10-31

**Authors:** Vanessa M. Conn, He Lin, Simon J. Conn

**Affiliations:** ^1^ Flinders Health and Medical Research Institute, College of Medicine & Public Health, Flinders University Bedford Park South Australia Australia

## 1 INTRODUCTION

Acute myeloid leukaemia, which is a haematological malignancy found in paediatric and adult individuals, carries the lowest mutational burden of any cancer type.^1^ A conserved genetic mutation found in over 70% of AML patients are the so‐called mixed lineage leukaemia (MLL) chromosomal translocations.^2^ Despite these oncogenic MLL fusions used in clinical diagnosis and representing a poor prognostic marker, which demands more intense therapeutic intervention, the molecular basis for this family of MLL mutations was unknown. A recent discovery has shed light on the pivotal role played by circular RNAs (circRNAs) in driving these MLL fusions in AML,^3^ offering novel insights that have the potential to transform clinical practice for leukaemia and beyond.

## 2 CIRCRNAS AS ENDOGENOUS RNA CARCINOGENS: A PARADIGM SHIFT

CircRNAs, a distinctive subclass of largely non‐coding RNA transcripts, have risen to prominence in recent years due to their circular, covalently‐closed structure, setting them apart from their linear RNA counterparts.^4^ Originally regarded as spurious byproducts of RNA splicing, circRNAs have transcended their humble origins to emerge as active and vital regulatory entities, implicated in various cellular processes. Notably, they have piqued the interest of leukaemia researchers owing to their capacity to bind DNA combined with plethora reports of their impact across all the hallmarks of oncogenesis.^5^

**FIGURE 1:**
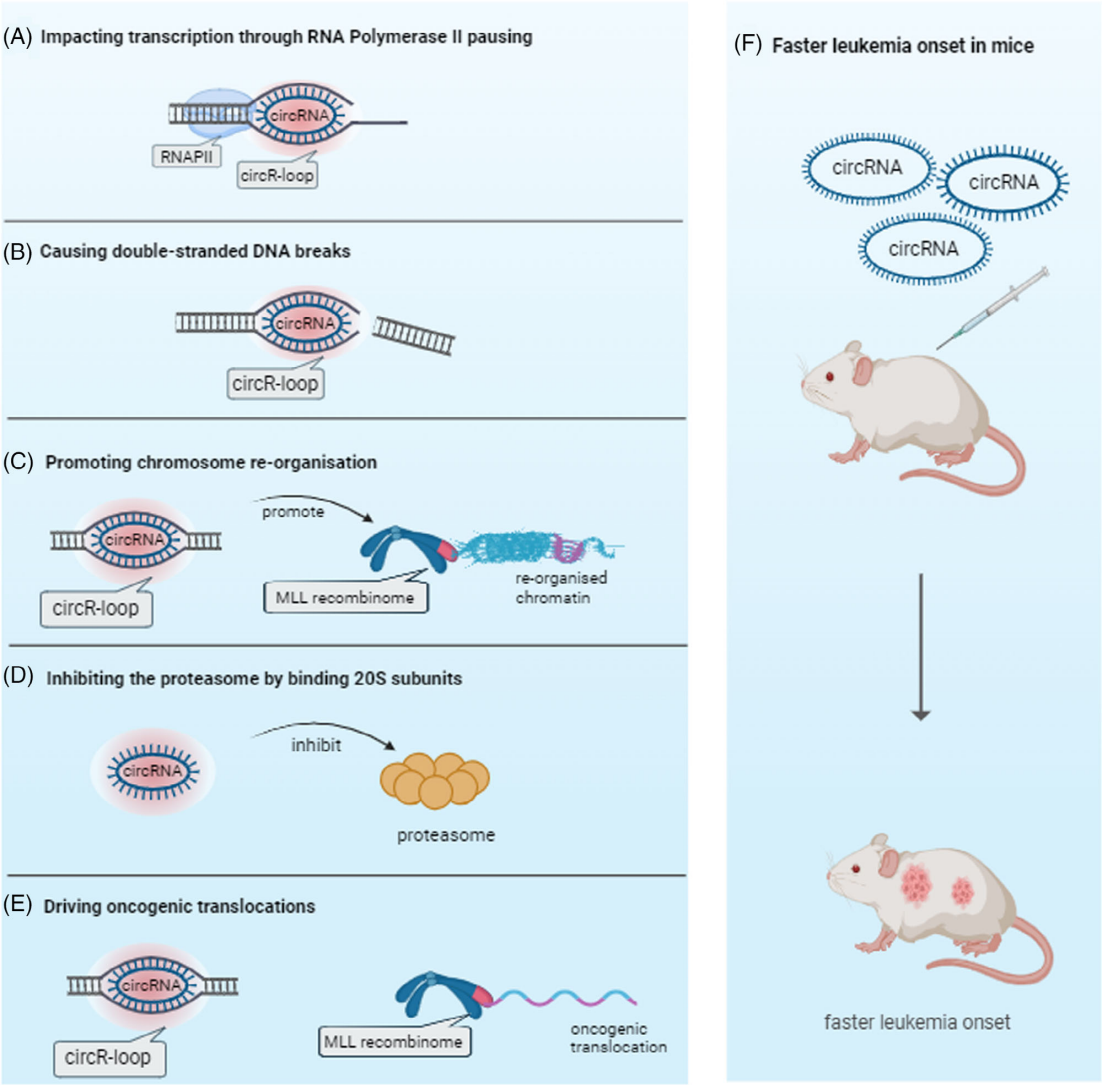
Functional consequences of circRNA:DNA hybrid (circR‐loop) formation within the MLL recombinome in leukaemia, in vitro and in vivo. Created with Biorender.com.

The discovery of circRNAs, such as circMLL(9,10), which are not merely bystanders but active participants in leukaemia, serves as a significant milestone in our comprehension of non‐coding RNAs' roles in cancer. One of the most striking findings in this research is the increased abundance of circMLL(9,10) in paediatric leukaemia compared with controls when quantified from neonatal blood spots (Guthrie cards) 1–7 years prior to disease onset.^3^ Furthermore, circMLL(9,10) and circRNAs from other genes, which MLL is known to fuse with, were found to bind to their own DNA locus (forming an R‐loop) corresponding with the MLL breakpoint cluster regions found in patients. The consequence of these R‐loops was site‐specific pausing of RNA polymerase II, inhibition of the proteasome, increased DNA breakage at these sites and three‐dimensional rearrangement of the genome bringing the MLL and fusion genes into closer proximity (Figure 1). Together, this resulted in specific fusions between MLL and their most common partner genes and resulted in earlier onset of leukaemia in two mouse models.

The ability of circMLL(9,10) to inhibit the proteasome signifies an intriguing facet of this newfound phenomenon. The proteasome, a cellular juggernaut responsible for protein degradation, is a focal point in the context of cancer biology. Inhibiting the proteasome catalyses the accumulation of proteins with the propensity to induce DNA breaks and chromosomal translocations, well‐acknowledged drivers of cancer.^6^ Proteasome inhibitors are widely used chemotherapeutics for multiple myeloma and other cancers, but circRNAs possess the ability to inhibit proteasome function, without killing the cell, but mutating the DNA. For the first time, this demonstrates circular RNAs into endogenous RNA carcinogens, in a process called endogenous RNA‐directed DNA damage (or ER3D). This challenges conventional paradigms, which predominantly emphasise external factors as drivers of DNA mutagenesis.

## 3 IMPLICATIONS AND FUTURE PROSPECTS OF CIRCULAR RNAS

**FIGURE 2:**
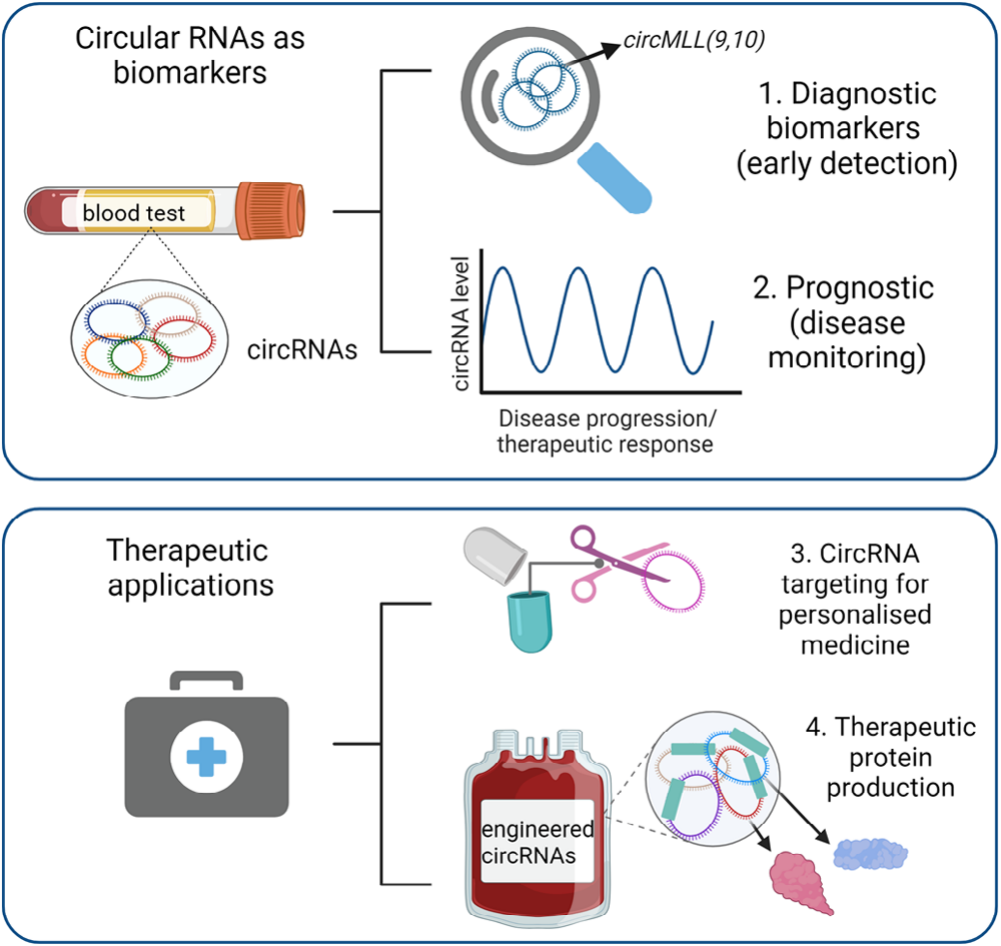
Potential uses of circular RNAs in cancer and human disease, including use as biomarkers (top panel) and as therapeutic targets (bottom panel). Created with Biorender.com.

This discovery raises a number of questions and research opportunities. These include do circRNAs shape other genetic rearrangements that could contribute to cancer or other diseases? Can we harness the potential of circRNAs for assisting patients? The most exciting prospect stemming from the interactions of circRNAs and leukaemia is the potential for clinical translation. circRNAs may revolutionise how we diagnose, prognosticate and treat paediatric leukaemia.^7^ Potential translational applications for circular RNAs are listed below (Figure 2):

1. *Biomarkers for early diagnosis*: The presence of these oncogenic circRNAs could serve as novel biomarkers for early leukaemia diagnosis. Profiling the abundance of specific circRNA signatures in patients' samples (liquid biopsy, e.g., blood, urine) might permit the detection of leukaemia at earlier stages, prior to symptom onset, particularly in paediatric populations where early intervention is paramount to boost patient survival.
2. *Prognostic indicators*: The abundance of specific circRNAs may aid in predicting disease progression and therapeutic response. In paediatric cases, where personalised treatment approaches are critical, circRNAs could guide physicians in stratifying patients into risk groups, ensuring tailored interventions. Changes in circRNA profiles throughout the course of treatment might provide real‐time feedback on how patients are responding to therapies, enabling physicians to adjust treatment strategies as needed. This would be particularly useful for cancers requiring costly imaging, or invasive procedures including brain or colorectal cancer.
3. *Personalised therapeutic targets*: By designing treatments that disrupt the oncogenic interactions between circRNAs and their targets (e.g., circMLL(9,10) and the proteasome), or that target the circRNA for destruction clinicians might offer more precise therapies with reduced side effects compared with standard frontline chemotherapies. Specifically, avoiding the use of topoisomerase inhibitors, like etoposide, that have been linked to recurrence of MLL‐rearranged leukaemias,^8^ may be warranted in cases where patients carry high levels of these oncogenic circRNAs.
4. *Engineering circRNA for enhanced protein production*: Their unique circular structure provides enhanced stability and resistance to degradation, making them promising candidates for the delivery of therapeutic proteins.^9^ Once introduced into cancer cells, these engineered circRNAs can carry specific targeting motifs and/or act as vehicles for the sustained expression of therapeutic proteins. This approach holds potential advantages over traditional gene delivery methods, as circRNAs can evade the host cell's natural degradation mechanisms and provide prolonged protein expression. Furthermore, by incorporating cancer‐specific promoters, circRNAs can be designed to deliver therapeutic proteins directly to tumour cells, reducing off‐target effects.

## 4 CONCLUSION

The revelation of circMLL(9,10) and its interplay with DNA, especially within the *MLL* gene, have ushered circRNAs into the forefront of leukaemia research and cancer research, at large. These circRNAs can no longer be considered passive transcripts but active participants in the intricacies of genetic instability and oncogenesis. Moreover, this finding may potentially shape future therapeutic landscapes to transform clinical practice in diverse malignancies. Indeed, a number of companies are undertaking clinical trials for therapeutic circRNAs in cancer and other human diseases.^9^ Timely integration of circRNAs into clinical practice as prognostic/diagnostic biomarkers and bona fide therapeutic targets could revolutionise the way we approach disease. Ultimately, this is another tool in the arsenal allowing doctors to provide more precise, personalised and effective care to improve outcomes and quality of life for children with formidable diseases, like cancer. This journey has now begun, and the potential is boundless.
